# PROMIS-deaf profile measure: cultural adaptation and psychometric validation in American sign language

**DOI:** 10.1186/s41687-020-00208-7

**Published:** 2020-06-09

**Authors:** P. Kushalnagar, R. Paludneviciene, M. Kallen, S. Atcherson, D. Cella

**Affiliations:** 1 Gallaudet University, Washington, DC, USA; 2 Northwestern University, Feinberg School of Medicine, Evanston, IL USA; 3 University at Arkansas for Medical Sciences, Little Rock, AR USA

## Background

The Patient Reported Outcomes Measurement Information System® (PROMIS®) is a structured collection of self-report measures that assess symptoms, functioning, and perceptions of health across a multidimensional spectrum of quality of life. PROMIS® instruments have been widely used in clinical research and practice [1–4]. In many applications of patient-reported outcome measurement, a combination of generic and targeted (e.g., disease-specific) measures is used, enabling comparable information across all groups measured while also capturing important and salient aspects of health status that might be specific to individual patient groups. Many PROMIS® instruments have been translated to other languages [5–8], using an iterative process of forward and back translation, independent reviews by bilingual experts, and pretesting on a population with characteristics similar to the target population [9]. There are currently over 2000 active research studies using PROMIS®. To date, however, PROMIS® instruments have not been available in American Sign Language (ASL) and have not been tested for validity in deaf and hard of hearing (DHH) adult populations. This is a medically underserved population that not only uses ASL but also has cultural values that are different from the mainstream. Factors that contribute to perceived DHH-specific quality of life outcomes have been found to differ from contributing factors associated with generic quality of life outcomes [10]. For this reason, patient-reported outcome measures should not only include generic but also DHH-specific items that assess a DHH person’s overall statuses and outcomes.

This paper describes the cultural adaptation, translation, test-retest administration, and psychometric methods used to make existing PROMIS® items and new communication items applicable to DHH people. We refer to this as the PROMIS®-Deaf Profile. We expect this measure to demonstrate that study participants with high Global Health status (better overall health) would have, on average, better PROMIS®-Deaf Profile domain-specific health status than those with low Global Health status (worse overall health).

## Methods

### Cultural adaption of existing items selected from PROMIS® item banks

After obtaining permission from PROMIS® Health Organization and with consultation from an advisory committee (AC) consisting of field experts, a total of 40 existing items were drawn from PROMIS® item banks and used as candidates for the PROMIS®-Deaf Profile. Because existing PROMIS® items were written for and validated in the US general population, we followed a specific cultural adaptation process to ensure that these items were relevant and could be answered by DHH individuals. The AC first reviewed and identified items that required adaptation for DHH individuals. A core cultural adaptation leadership team then replaced specific language or colloquial wordings (e.g., “listen” was changed to “pay attention”) and presented all revised items to the full AC for review and approval prior to linguistic translation.

### Development of new communication health and early life communication experience items

Using the grounded theory approach to item generation [11], communication health items were developed for the DHH population to assess perceived quality of life specific to being DHH. We used transcripts from in-depth interviews conducted in a previous qualitative study [10] to draft 84 items and then selected additional items from the existing literature to generate an item pool. Each item was evaluated for measure inclusion on the basis of meeting the following two criteria: 1) the item should measure the domain construct of “Communication Health,” and 2) the item should be relevant to the DHH experience, regardless of hearing level or other DHH-specific characteristics. Nineteen items were final candidates for the Communication Health domain of the PROMIS®-Deaf Profile. With permission, four items were modified from the Youth Quality of Life-DHH Module [11] to allow for retrospective reporting of early life communication experiences (ELCE) by DHH adults. The significance of early life communication experiences is highlighted in several studies that have reported its connection with quality of life outcomes in DHH adults [12–14].

### Linguistic validation of PROMIS®-deaf profile items in ASL

The ASL-English bilingual translation team consisted of two forward translation consultants and a backward translation consultant. All were bilingual in ASL and English, and all had previous experience translating test items. Near-final PROMIS®-Deaf Profile items in ASL were next carefully evaluated by the project director to ensure their conceptual equivalence to the original items in English. The ASL version of the PROMIS®-Deaf Profile was digitally recorded by a native ASL signer with over 10 years of acting experience and teaching ASL linguistics. In addition, two deaf research members with clinical psychology and assessment backgrounds coached the ASL signer during videotaping, to ensure the highest linguistic fidelity to item content.

Using methods from the US National Center for Health Statistics Cognitive Survey Laboratory [15, 16], cognitive debriefing sessions were used to assess 1) whether DHH participants found the signed items easy to complete; 2) if they found any problems with the signed translations; and 3) what their overall reaction was to the ASL version of the PROMIS®-Deaf Profile. We also assessed comprehension by observing consistency in responses to the questions. In two waves of face-to-face interview sessions with four-to-five ASL signers per wave, most participants had a high school education or less. In all cognitive interview sessions, “no English” version items (i.e., ASL only, without any English supporting text) were shown, so as to focus on the clarity of ASL content delivery. If a participant had difficulties with understanding any signing, the interview team tested alternative ASL translations or asked the participant to propose ideas to improve clarity of the items, including replacing signs or phrases. These participant-engagement procedures helped improve the face validity of the items in ASL.

### Psychometric testing

#### Participants

After the Gallaudet University Institutional Review Board approved the study protocol, we recruited participants from the DHH community across the USA, including Hawaii and Alaska. Multiple recruitment methods were employed: snowball sampling through personal networks, distributing flyers, and advertising on DHH-centered organization websites and e-newsletters. A total of 1717 individuals who signed up met eligibility for early deafness (born or became DHH before 13 years old), having bilateral hearing loss, and using ASL in their daily communication. We then enrolled those who provided consent (*N* = 1612). Some 258 consented participants did not complete the demographics and survey items. Thus, the final psychometric sample included 1354 adult participants.

### Study measures

#### Existing PROMIS® domains and new PROMIS®-deaf profile domains

Existing domains in the PROMIS®-Deaf Profile included Anger (2 items), Anxiety (4 items), Depression (4 items), Fatigue (10 items), Global Health (10 items), Social Isolation (2 items), and Social Support (6 items). New domains were also added to the PROMIS®-Deaf Profile: Communication Health (19 items) and Early Life Communication Experiences (ELCE-4 items).

#### Demographic and clinical variables

DHH study participants were asked to answer questions about their demographic background and medical conditions. Medical conditions were used to assign clinical and non-clinical status to assess the clinical sensitivity of the PROMIS®-Deaf Profile. Clinical status was assigned if the respondent confirmed one or more medical conditions and reported severity in symptoms associated with the medical condition(s).

### Measure administration and data collection

The PROMIS®-Deaf Profile measures were configured as fixed-length assessments and administered via the study’s secure website between April 2016 and April 2018. Study participants accessed this protected website using research study, personal, or publicly-available computers, each of which required internet access. Participants were typically able to complete their assigned measure assessments within 7 days of initial assignment.

#### “ASL-only” survey administration

For the web-based survey application, supporting English text was hidden. By having all respondents view items and response options only in ASL, we were able to establish the item administration condition that all ASL-Only survey participants view each item only in ASL before providing responses.

#### “ASL + English” survey administration

Respondents in this subsample could see both ASL video and supporting English text. The ASL + English survey was employed for the targeted purpose of investigating each measure’s potential for bias if supporting English text were included along with each ASL video. The inclusion criteria and recruitment procedures for the ASL + English survey administration were identical to those described for the ASL-Only survey administration.

### Psychometric analyses

We employed extensive psychometric analyses, following those recommended in PROMIS®‘s guidelines and standards for measure development and evaluation [17]; analyses included confirmation of the unidimensionality assumption for existing PROMIS® measures and new DHH-specific measures and support for unidimensional item response theory (IRT) model fitting and item calibration. We determined our study’s required sample size on the basis of it being large enough to conduct (a) robust unidimensional confirmatory factor analysis (CFA), (b) two-levels-per-factor differential item functioning (DIF) studies, and, where required, (c) accurate estimation of new IRT item parameters, from which individual participant scores could be estimated.

First, for the new DHH-specific domain, Communication Health, we conducted exploratory factor analysis (EFA) to better understand its dimensionality and, thus, have evidence at hand to propose unidimensional factors and their associated items to undergo further measure development and evaluation. Then, for all PROMIS-Deaf Profile measures, existing and new, we obtained classical test theory (CTT)-based assessments of item and measure performance, including estimates of item-adjusted total score correlations and analyses of potential total score floor and ceiling effects. Next, for each PROMIS-Deaf Profile measure that was four or more items in length, we fit item response data to a unidimensional confirmatory factor analysis (CFA) model to evaluate item and overall CFA model fit. Finally, for each new PROMIS-Deaf Profile measure that was four or more items in length, we fit item response data to an item response theory (IRT)-based graded response model (GRM) to (a) evaluate item and overall GRM model fit and (b) calibrate the items. Further supporting analyses were conducted, included reliability studies (internal consistency and test-retest), bias studies (differential item functioning by item presentation method, age, education, and gender factors), and validity studies (concurrent and known groups).

#### CTT-based assessments

To evaluate item performance, we identified items (a) with sparse response cells (i.e., items having one or more response categories used by < 10 respondents) and (b) whose item-adjusted total score correlation (i.e., the item’s correlation with its source measure’s total score adjusted by excluding the item correlated) was < 0.40. To evaluate measure performance, we (a) studied measure floor and ceiling effects (i.e., if ≥10% of respondents had the minimum or maximum scores possible per measure) and (b) examined measure score distributions for skewness, excess kurtosis, and the extent to which the distributions could be considered normal. Finally, we calculated descriptive statistics (i.e., mean, median, standard deviation, minimum, and maximum score) for each measure. We conducted CTT-based analyses in IBM SPSS (version 25) [18].

#### CFA-based assessments

We assessed each PROMIS-Deaf Profile measure that was four or more items in length (i.e., Anxiety, Depression, Fatigue, Global Health-Mental, Global Health-Physical, Social Support, Communication Health, and ELCE) for essential unidimensionality. To evaluate item performance, we identified items (a) with low factor loadings (i.e., < 0.50) or (b) with evidence suggesting local dependence (i.e., a residual correlation > 0.20 or a correlated error modification index ≥100). To evaluate measure performance, we estimated a single-factor CFA model per measure to obtain evidence supportive of measure essential unidimensionality. We assessed overall model fit to each measure’s item response data using the following recommended fit criteria: Comparative Fit Index (CFI) ≥ 0.95, Tucker-Lewis index (TLI) ≥ 0.95, root mean square error of approximation (RMSEA) < 0.10, and standardized root mean residual (SRMR) < 0.08 [19–24]. We conducted CFA-based analyses in Mplus (version 7.4).

#### Measure reliability studies

We estimated two types of reliability for all PROMIS-Deaf Profile measures: internal consistency reliability and test-retest reliability. For internal consistency reliability, we estimated Cronbach’s alpha and reported its raw score value for each measure. For test-retest reliability, we estimated the intra-class correlation (ICC) coefficient using a subset of *n* = 100 ASL-Only Subsample study participants who had completed a second set of assigned measures within seven to 10 days of completing their first assigned measures. For all PROMIS-Deaf Profile measures, we obtained ICC values based on (a) systematic error plus random error and (b) random error only. For both internal consistency and test-retest reliability estimate standards, we used reliability ≥0.70 as the criterion for using a measure to make group-level comparisons and reliability ≥0.90 as the criterion for using a measure to make individual-level comparisons. We conducted reliability analyses in IBM SPSS (version 25) [18].

#### DIF studies

We assessed each PROMIS-Deaf Profile measure that was four or more items in length (i.e., Anxiety, Depression, Fatigue, Global Health-Mental, Global Health-Physical, Social Support, Communication Health, and ELCE) for differential item functioning (DIF), which is a potentially biasing item performance effect. Items exhibiting DIF measure investigated population subgroups differently (i.e., unequally). For example, males at measured-trait level Y might consistently receive higher positive Item X scores than do females who are at the same measured-trait level Y and who respond to the same Item X. In Phase One of our DIF studies, identifying items potentially exhibiting DIF, we employed a hybrid IRT ability score and ordinal logistic regression framework, as implemented in the R package, “lordif” (Version 0.3–3) [25]. For these analyses we used a Nagelkerke pseudo-R2 change ≥0.02 as our criterion for flagging items with potential DIF. In Phase Two of these analyses, to determine if flagged DIF items meaningfully impacted measure scores per population subgroups, we used as our criterion for measure score DIF impact > 2% of DIF-corrected vs. DIF-uncorrected score differences exceeding uncorrected score standard errors. We investigated the following factors for potential DIF: item presentation method, age, education, and gender. We conducted DIF analyses in R (version 3.23) [26].

#### Preliminary validation of PROMIS-deaf profile measures

To establish initial evidence supporting the validity of the PROMIS-Deaf Profile measures, we obtained estimates of convergent and discriminant validity and known-groups validity.

#### Convergent and discriminant validity

To evaluate the convergent and discriminant validity of the PROMIS-Deaf Profile measures, we estimated Pearson’s r and Spearman’s rho correlations (to account for potentially non-normal measure score distributions) (a) among all PROMIS-Deaf Profile domain-specific measures (i.e., Anger, Anxiety, Depression, Fatigue, Social Isolation, Social Support, Communication Health, ELCE) and (b) between domain-specific vs. general health status measures (i.e., Global Health-Mental, Global Health-Physical). We interpreted the magnitude of the absolute value of a Pearson’s r or Spearman’s rho between measure scores as follows: From 0.60 to 1.00 is a “strong” correlation; from 0.30 to < 0.60 is a “moderate” correlation; and from 0.00 to < 0.30 is a “weak” correlation [27].

#### Known-groups validity

To evaluate known-groups validity of the PROMIS-Deaf Profile measures, we divided our study participants into approximate quartiles, based first on their Global Health-Mental scores and based second on their Global Health-Physical scores. Via analysis of variance (ANOVA), we then compared the PROMIS-Deaf Profile domain-specific measure scores (i.e., Anger, Anxiety, Depression, Fatigue, Social Isolation, Social Support, Communication Health, ELCE) of participants with the lowest quartile Global Health-Mental scores (i.e., raw scores from 4 to 12) vs. those with the highest quartile Global Health-Mental scores (i.e., raw scores from 16 to 20). We conducted a similar set of ANOVA-based PROMIS-Deaf Profile measure score comparisons between participants with the lowest quartile Global Health-Physical scores (raw scores 4 to 13) vs. those with the highest quartile Global Health-Physical scores (raw scores 17 to 20). For all of these comparisons, we hypothesized that study participants with high quartile Global Health status (i.e., better overall mental or physical health) would have, on average, better PROMIS-Deaf Profile domain-specific health status than those with low quartile Global Health status (i.e., worse overall mental or physical health).

Finally, we compared all PROMIS-Deaf Profile measure scores of our study participants by select demographic (i.e., age, gender, level of education, race/ethnicity, preferred language) and clinical variables (current hearing level, functional hearing status, when hearing loss occurred, if hearing loss is clinical/non-clinical, if family background includes DHH members). For all ANOVAs, we used an F test *p* value < 0.05 to indicate statistically significant group differences, and we calculated Cohen’s D values to report group mean difference-based effect sizes. We conducted these ANOVA validity analyses in IBM SPSS (version 25) [18].

#### Sample size requirements

We determined our study’s required sample size on the basis of it being large enough to conduct (a) robust unidimensional CFA analyses, (b) two-levels-per-factor DIF studies, and, as required, (c) accurate estimation of new IRT item parameters, from which individual participant scores could be estimated. For CFA analyses and GRM-based IRT parameter estimation, sample size recommendations range from *N* = 200 to 1000 (ref) or a minimum of 5 to 10 individuals per item. For DIF studies employing an IRT-based ability score estimate, a minimum sample size of *N* = 500, with a minimum of *n* = 200 participants per factor level within each DIF factor tested, is required [28, 29]. Therefore, we required a minimum *n* = 500 ASL-Only Subsample study participants and a minimum n = 200 ASL + English Subsample study participants to appropriately conduct our planned item presentation method DIF analyses. If this initial DIF study (i.e., ASL-Only vs. ASL + English) were to indicate meaningful item presentation method DIF impact, we would need to be prepared to proceed with PROMIS-Deaf Profile measure development using distinct ASL-Only- and ASL + English-specific samples. For our additional DIF studies, we anticipated our obtained sample would allow us to compare study participants by age (≤40 years vs. > 40 years), education (≤ high school degree vs. > high school degree), and gender (male vs. female).

## Results

Our study sample was composed of *N* = 1354 participants (mean age = 42; SD = 16). Just over half (57%) were female, and 65% were White. More than half the sample (62%) were born DHH, with 71.5% reporting severe or profound hearing loss. About 45% of the sample preferred using sign language as their primary language, and 42% did not have a college degree. Table 1 presents clinical status across gender and age groups.

**Table 1:** Unweighted Sample Characteristics by Clinical Status

Characteristics	Non Clinical	Clinical
Gender	n (%)	
Male	341 (44.9)	173 (33.4)
Female	410 (54.0)	329 (63.5)
Non-Binary	8 (1.1)	16 (3.1)
Subtotal^a^	759	518
Age	n (%)	
18–31	274 (36.0)	148 (28.4)
32–48	275 (36.1)	166 (31.8)
48–95	212 (27.9)	208 (39.8)
Subtotal^a^	761	522
Race	n (%)	
White	499 (66.4)	359 (70.1)
Non-White	253 (33.6)	153 (29.9)
Subtotal^a^	752	512
Education	n (%)	
High school or lower	136 (18.4)	124 (24.6)
Some college	157 (21.2)	116 (22.8)
College degree	446 (60.4)	267 (52.6)
Subtotal^a^	739	508
U.S. region	n (%)	
Northeast	88 (11.9)	73 (14.3)
South	261 (35.3)	189 (37.1)
Midwest	141 (19.1)	91 (17.9)
West	250 (33.8)	156 (30.6)
Subtotal^a^	740	509
Preferred language	n (%)	
ASL Only	353 (46.8)	222 (42.7)
ASL and English	402 (53.2)	298 (57.3)
Subtotal^a^	755	520
Deaf identity	n (%)	
Deaf	530 (77.9)	350 (74.5)
Hard of Hearing	150 (22.1)	120 (25.5)
Subtotal^a^	680	470
Functional hearing	n (%)	
Can understand all or most	207 (28.4)	171 (34.0)
Can understand some or little	314 (43.2)	219 (43.5)
Cannot understand at all	206 (28.3)	114 (22.6)
Subtotal^a^	727	504
Listening device	n (%)	
Do not use	405 (59.5)	274 (59.1)
Hearing aids	225 (33.0)	153 (33.0)
Cochlear implants	47 (6.9)	36 (7.8)
Other types	4 (0.6)	1 (0.2)
Subtotal^a^	681	464

^a^Missing responses are not included in the subtotal

Subsamples of *n* = 614 and *n* = 740 participants completed ASL-only or ASL + English versions of the PROMIS®-Deaf Profile, respectively. Results from DIF by item presentation method studies did not identify item performance differences in any PROMIS®-Deaf Profile measures, using our hybrid logistic regression and IRT-based item-flagging criterion. The two subsamples characterized by item presentation method were therefore pooled and analyzed together.

For classical test theory-based item performance among the existing PROMIS® measures, we identified one item whose item-adjusted total score correlation was < 0.40 (Global Health-Physical item: “How would you rate your fatigue on average?”). All existing PROMIS® measures met the proposed CFA modeling CFI and SRMR fit criteria, and all but one met RMSEA and TLI fit criteria. The one existing PROMIS® measure that did not meet the proposed RMSEA or TLI fit criteria was Global Health-Physical (RMSEA = 0.13; TLI = 0.90). Overall, individual item function and overall measure fit statistics provided evidence highly supportive of each modeled measure’s essential unidimensionality. Table 2 lists final items that are psychometrically acceptable for inclusion in the PROMIS®-Deaf Profile. For the 19-item Communication Health domain, eight modeled items met proposed CFA essential unidimensionality fit criterion (i.e., RMSEA = .08; CFI = 0.96; TLI = 0.95; SRMR = 0.04). Our exploratory factor analysis (EFA) of the four ELCE items provided results suggestive of multi-dimensionality; subsequent CFA analyses confirmed a lack of acceptable fit to a unidimensional model (i.e., RMSEA = .40; CFI = 0.94; TLI = 0.82; SRMR = 0.10). These CFA analyses showed two item pairs had highly correlated error values (i.e., a sign of local dependence). We, therefore, divided the four ELCE items into two 2-item sets before proceeding with subsequent psychometric analyses. The 2-item sets are named as follows: ELCE-Direct Child-Caregiver Communication (i.e., ELCE-Direct CCC) and ELCE-Indirect Family Communication and Inclusion (i.e., ELCE-Indirect FCI).

**Table 2:** Existing and New PROMIS-Deaf Profile Domains and Item Content

**Existing PROMIS Domain**	**Item ID**	**Item Content**
Anger	EDANG15	I felt like I was ready to explode
	EDANG09	I felt angry
Anxiety	EDANX01	I felt fearful
	EDANX40	I found it hard to focus on anything other than my anxiety
	EDANX41	My worries overwhelmed me
	EDANX53	I felt uneasy
Depression	EDDEP04	I felt worthless
	EDDEP06	I felt helpless
	EDDEP29	I felt depressed
	EDDEP41	I felt hopeless
Fatigue	FATEXP20	How often did you feel tired?
	FATEXP42	How much mental energy did you have on average?
	FATEXP48	How often did you find yourself getting tired easily?
	FATIMP56	How often were you too tired to socialize with your friends?
	FATIMP53	How often were you too tired to take a short walk?
	FATIMP25	How often was it an effort to carry on a conversation because of your fatigue?
	FATIMP13	How often were you too tired to do errands?
	FATIMP26	How often were you too tired to socialize with your family?
	FATEXP18	How often did you run out of energy?
	FATEXP26	How often were you too tired to enjoy life?
Global Health	Global01	In general, would you say your health is …
	Global02	In general, would you say your quality of life is …
	Global03	In general, how would you rate your physical health...
	Global04	In general, how would you rate your mental health, including your mood and your ability to think?
	Global05	In general, how would you rate your satisfaction with your social activities and
	Global06	To what extent are you able to carry out your everyday physical activities such as walking, climbing stairs, carrying groceries, or moving a chair?
	Global07	In the past 7 days, how would you rate your pain on average?
	Global08	In the past 7 days, how would you rate your fatigue on average?
	Global09r	In general, please rate how well you carry out your usual social activities and roles. (This includes activities at home, at work and in your community, and responsibilities as a parent, child, spouse, employee, friend, etc.)
	Global10	In the past 7 days, how often have you been bothered by emotional problems such as feeling anxious, depressed or irritable?
Social Health-Support	FSE31053x2d	I have someone who will pay attention to me when I need to discuss something.
	FSE31059x2d	I have someone to confide in about my problems
	SS12x	I have someone who makes me feel appreciated
	SSQ3x2d	I have someone to go to when I have a bad day
	FSE31069x2	I have someone who understands my problems
	SSECaPS6d	I have someone I trust to share my feelings with.
**New PROMIS-Deaf Profile Domain**	**Item ID**	**Item Content**
Communication Health	Comm03	I feel my life is good even though I am deaf or hard of hearing.
	Comm07	I am satisfied with the way I communicate with deaf/hh people
	Comm13	In general, I feel people accept me as a person who is deaf or hard of hearing.
	Comm15	People who are close to me accept me as a person who is deaf or hard of hearing.
	Comm16	I am able to easily get the information I need to make decisions
	Comm17	People closest to me share the same communication and language.
	Comm18	People close to me make an effort to communicate with me because I am deaf or hard of hearing.
	Comm19	I have friends who are like me and can understand what it is like to be deaf or hard of hearing.
Early Life Communication Experiences (ELCE)	ELCE03	Thinking about the person/caregiver/parent who took care of you the most when you were growing up, how much did this person understand you?
	ELCE04	Thinking about the person/caregiver/parent who took care of you the most when you were growing up, how much did you understand this person?
	ELCE05	When you were growing up, how often did you feel included in family conversations or discussions?
	ELCE06r	When you were growing up, how often did you feel ignored or left out by your family?

For Cronbach’s alpha internal consistency reliability, all new PROMIS®-Deaf Profile measures met the reliability ≥0.70 criterion for using a measure to make group-level comparisons (i.e., for Communication Health, ELCE-Direct CCC, and ELCE-Indirect FCI, alpha = .81, .84, and .75, respectively). Two existing PROMIS® measures did not meet the ≥0.70 reliability criterion: Global Health-Physical (.66) and Social Isolation (.58). For test-retest reliability, estimated using intraclass correlation (ICC) coefficients based on both random error plus systematic error and random error only, all PROMIS®-Deaf Profile measures met the reliability ≥0.70 criterion.

### Preliminary validation of the PROMIS®-deaf profile

For convergent and discriminant validity of the PROMIS®-Deaf Profile, we correlated the two general health status measures vs. the domain-specific measures: Correlations were low to moderate in magnitude (see Table 3). Known-group validity results from ANOVAs conducted indicated that members of the highest quartile Global Health-Mental and Global Health-Physical groups had statistically significantly (*p* < 0.05) “better” or “healthier” domain status scores for all PROMIS-Deaf Profile measures tested than did members of the lowest quartile groups. Thus, known-groups validity results supported our research hypothesis: Study participants with high Global Health status (better overall health) would have, on average, better PROMIS®-Deaf Profile domain-specific health status than those with low Global Health status (worse overall health). Additionally, posthoc analysis indicated similar observations for Communication Health, ELCE-Direct CCC, and ELCE-Indirect FCI, with high status performing better on PROMIS-Deaf Profile domain-specific health status than those with low Communication Health/ELCE statuses (worse DHH-specific health).

**Table 3:** Spearman Correlations

	Anger	Anxiety	Depression	Fatigue	Social Isolation	Social Support	Global Health-Mental	Global Health-Physical	Communication Health	ELCE-Direct CCC
**Anxiety**	0.50									
**Depression**	0.56	0.71								
**Fatigue**	0.40	0.56	0.58							
**Social Isolation**	0.30	0.44	0.46	0.42						
**Social Support**	−0.23	−0.16	−0.25	−0.21	−0.24					
**Global Health-Mental**	−0.38	−0.56	−0.59	−0.52	−0.42	0.26				
**Global Health-Physical**	−0.31	−0.42	−0.43	− 0.56	− 0.28	0.21	0.57			
**Communication Health**	−0.25	− 0.22	− 0.29	− 0.24	−0.34	0.42	0.32	0.25		
**ELCE-Direct CCC**	−0.14	− 0.12	−0.20	− 0.16	−0.15	0.27	0.09	0.13	0.32	
**ELCE-Indirect FCI**	−0.16	− 0.16	−0.18	− 0.20	−0.22	0.17	0.17	0.19	0.27	0.51

#### PROMIS®-deaf profile mean score differences by clinical characteristics

“Non-clinical” DHH subsample participants had statistically significantly (*p* < 0.05) “better” or “healthier” domain status mean scores for all PROMIS-Deaf Profile measures tested than did “clinical” DHH subsample participants.

## Discussion

We have described the development and initial validation of the PROMIS®-Deaf Profile assessments of common and unique symptoms experienced by the DHH community in the US. We administered this profile to a national sample of DHH adults and report here our psychometric findings. This work brings to the DHH community a set of patient-reported outcome measures that allow for comparison to other populations and identifies unique measures of concepts important to DHH individuals. Major strengths of the PROMIS®-Deaf Profile are its large psychometric sample, accessibility in ASL and English, suitability for use across diverse DHH subgroups, and the addition of new domains that tap into key dimensions of communication health and early life communication experiences relevant to a DHH person’s quality of life outcomes.

The early life communication experiences domain has two robust factors; the resulting assessments each showed good reliability. These factors (ELCE-Direct Child-Caregiver Communication and ELCE-Indirect Family Communication and Inclusion) can be used to assess adverse childhood communication experiences that may impact a DHH person’s quality of life outcomes. The Communication Health domain can be used to assess a DHH patient’s perception of current quality of life and outcomes specific to being DHH. This domain was found to be distinct from the generic patient-reported outcomes assessed through PROMIS® Global Health and other domains.

The PROMIS®-Deaf Profile demonstrates validity for use among diverse DHH individuals, now available in ASL with English text for added support. Test-retest and internal consistency reliability, as well as clinical sensitivity between clinical and nonclinical groups were all, overall, quite good. Limitations include not evaluating for measurement invariance across time and establishing clinically important differences, both of which would need to be assessed in a future clinical study.

## Data Availability

The dataset generated during the current study are not publicly available due to the nature of the small community and the need to protect its confidentiality.

## Abbreviations

PROMIS®: Patient Reported Outcomes Measurement Information System®
DHH: Deaf/hard-of-hearing
AC: Advisory committee
ASL: American Sign Language
PRO: Patient reported outcomes
ELCE: Early life communication experiences
IRT: Item response theory
CFA: Confirmatory factor analysis
